# Discrete and differentiated encoding of distinct components of spatial experience occurs within the proximodistal subfields of the dorsoventral axis of the hippocampus

**DOI:** 10.3389/fnbeh.2026.1895371

**Published:** 2026-08-07

**Authors:** Muxin Lin, Thu-Huong Hoang, Denise Manahan-Vaughan

**Affiliations:** 1 Department of Neurophysiology, Medical Faculty, Ruhr University Bochum, Bochum, Germany; 2 International Graduate School of Neuroscience, Ruhr University Bochum, Bochum, Germany

**Keywords:** dorsoventral, fluorescence *in situ* hybridization, hippocampus, immediate early gene, rat, spatial learning

## Abstract

A multitude of studies have confirmed the essential role of the hippocampus in the acquisition and updating of associative experience. In rodents, spatial memories are dependent on hippocampal information processing and encoding. The role of the dorsal hippocampal pole is well documented, but information about the roles of the intermediate and ventral hippocampus in spatial learning are few and often contradictory. Here, we used fluorescence *in situ* hybridization (FISH) to identify nuclear expression of the immediate early (IEG) gene, Homer1a, that was triggered by specific spatial learning events in hippocampal neurons of adult male rats. We compared IEG expression in the cornu ammonis (CA) and dentate gyrus (DG) along the proximodistal and dorsoventral hippocampal axis, triggered by novel learning about spatial constellations of large (macroscale), or partially concealed (microscale) objects within a familiar environment. We observed that the dorsal hippocampus predominates with regard to the neuronal encoding of both forms of spatial content information, whereby the distal CA1 (dCA1) and proximal CA3 (pCA3) preferentially encode microscale spatial location. The infrapyramidal blade of the DG, as well as pCA3, encode macroscale information. The intermediate CA shows increased IEG expression in dCA1 triggered by novel microscale information, but the DG of neither the intermediate, nor ventral, hippocampus encode macroscale information. The ventral hippocampus exhibits IEG expression patterns that are unique in comparison to the other two structures: here, both CA1 (proximal and distal) and pCA3 encode macroscale information. Taken together, these findings suggest that the intermediate and ventral hippocampus support spatial memory encoding, but may do so in support of differentiated and distinct aspects of associative learning.

## 1 Introduction

The hippocampus is essential for the acquisition and updating of declarative and, most especially, episodic memories (Tulving and Markowitsch, 1998; Sugar and Moser, 2019) that typically derive from multisensory experience. In rodents, this kind of memory is scrutinized in studies of associative memories, ranging from studies of hippocampal participation in spatial representations (Jeffery, 2024) and long-term spatial memory (Hagena and Manahan-Vaughan, 2024), through associative fear conditioning (Chaaya et al., 2018) and episodic-like memory (Asiminas et al., 2022; Hagena and Manahan-Vaughan, 2024; Zentall, 2026). Most functional studies of hippocampus-dependent memory have been conducted in the dorsal hippocampus. However, distinct molecular expression patterns have been identified along the dorsoventral axis of the hippocampus pointing toward genetic domains with clearly demarcated borders within this axis (Thompson et al., 2008; Strange et al., 2014). Moreover functional delineations in dorsal, intermediate and ventral hippocampal parts have been derived from behavioral (Bast et al., 2009; Loureiro et al., 2012), electrophysiological (Papatheodoropoulos and Kostopoulos, 2000; Kenney and Manahan-Vaughan, 2013; Dubovyk and Manahan-Vaughan, 2018), and molecular studies (Pandis et al., 2006; Maggio and Segal, 2009; Dubovyk and Manahan-Vaughan, 2018).

In fact, it has long been a matter of debate as to whether the dorsal and ventral poles of the hippocampus are distinct in their cognitive functions. The conservative view is that the dorsal hippocampus is responsible for cognitive functions, comprising for example, spatial memory in rodents, whereas the ventral hippocampus processes state-dependent (stress, emotions, affect) information (Fanselow and Dong, 2010). However, a more liberal interpretation considers that both hippocampal poles can engage in both kinds of information processing, albeit with putatively different cognitive outcomes (Contreras et al., 2018; Yu et al., 2018; Ramos and Morón, 2022; Wu et al., 2024; Yoon et al., 2024). Moreover, recent studies suggest that functional interactions between the dorsal and ventral poles are required for efficacious associative learning (Lee et al., 2019; Avigan et al., 2020).

Between the poles of the hippocampus lies the intermediate part, and this structure has also been ascribed functions that are arguably distinct from the dorsal and ventral poles. These include rapid experience encoding (Bast, 2007; Bast et al., 2009; Jarrard et al., 2012) and differences in synaptic plasticity responses to spatial contexts (Kenney and Manahan-Vaughan, 2013). Although all three dorsoventral regions possess the same canonical architecture of the trisynaptic circuit, as well as the same interconnectivity, along with inputs from the entorhinal cortex (Andersen et al., 1971; Dolorfo and Amaral, 1998b; Dolorfo and Amaral, 1998a; Witter et al., 2000), they also display distinct efferent and afferent interconnectivity. For example, the dorsal CA2 region receives inputs from the paraventricular nucleus (Althammer et al., 2022) and projects unidirectionally to ventral CA1 (Meira et al., 2018). The ventral hippocampus is unique in its reciprocal connectivity with the amygdala (Felix-Ortiz and Tye, 2014; Ritger et al., 2024). Moreover, although both the intermediate and the ventral hippocampus project to the prefrontal cortex, their afferent routes differ (Takita et al., 2013).

The dentate gyrus serves as one hippocampal recipient of entorhinal cortex (EC) inputs. Moreover it can be subdivided into further compartments including a suprapyramidal blade (sDG), an infrapyramidal (iDG) blade, and the hilus (Wyss, 1981; Amaral and Witter, 1989; Tamamaki, 1997). Mossy fiber axons from both DG blades to the cornu ammonis share similar properties (West et al., 1981) and project to the superficial pyramidal cells of CA3 (Claiborne et al., 1986). However, the infrapyramidal blade also sends axons into the deep pyramidal layers of this subfield (Claiborne et al., 1986). Furthermore, the same study also reported that, although both the sDG and iDG (with the exception of the tip of sDG) send mossy fibers to the proximal region of CA3 (pCA3), only the sDG tip projects to the distal CA3 (dCA3). These findings align with later studies that reported that iDG and sDG process spatial information differently (Chawla et al., 2005; Hoang et al., 2018). In addition, along with anatomical documentation (Ishizuka et al., 1990; Witter et al., 2000; Witter, 2007), it has been reported that the proximal and distal components of the CA3 and CA1 regions of the dorsal hippocampus process and store different components of spatial experience (Sauvage et al., 2013; Hoang et al., 2018; Hoang and Manahan-Vaughan, 2025).

The “two streams” hypothesis proposes that item features (“what” information) are conveyed via the perirhinal cortex and lateral EC to the hippocampus, while spatial context (“where” information) is processed via the postrhinal cortex and medial EC, before reaching the hippocampus via the medial perforant path (Mishkin et al., 1983; Fyhn et al., 2004; Hargreaves et al., 2005; Furtak et al., 2007). This concept is supported by functional studies revealing that the distal CA1 (dCA1) and pCA3 regions of the dorsal hippocampus preferentially encode item features, whereas the proximal CA1 (pCA1) and dCA3 regions are specialized in spatial processing (Henriksen et al., 2010; Burke et al., 2011; Ito and Schuman, 2012; Sauvage et al., 2013). This kind of information processing facilitates subregion-specific expression of hippocampal synaptic plasticity, which strikingly, can occur by means of monosensory, or multisensory, information encoding (Kemp and Manahan-Vaughan, 2008b; Hagena and Manahan-Vaughan, 2011; André and Manahan-Vaughan, 2013; Dietz and Manahan-Vaughan, 2017).

Rodents encode visual cues during exploration and navigation in different ways depending on the size of the objects, their view perspective, and whether the rodent is object-naive, or has experienced the object in the past (Chan et al., 2012; Scaplen et al., 2014). Spatial content can be differentiated into directional cues (i.e., derived from landmarks, LM), which can be used as waypoints for navigation, or allocentric anchors (‘where’ information), and more subtle local cues, which provide information about the topography or specific sensory content of the environment (‘what’ information). Acquisition of these kinds of spatial information drive the expression of hippocampal synaptic plasticity and induce neuronal immediate early gene (IEG), albeit in a subfield-specific manner: Exposure to novel LM constellations facilitates long-term depression (LTD) in the dorsal DG and mossy fiber-CA3 synapses (Kemp and Manahan-Vaughan, 2008b; Hagena and Manahan-Vaughan, 2011) and increases nuclear immediate early gene expression in neurons of the dorsal proximal CA3 and DG regions (Hoang et al., 2018). Exposure to novel spatial constellations of partially concealed visuospatial information facilitates hippocampal LTD in the dorsal CA1 region and associational-commissural CA3 synapses (Kemp and Manahan-Vaughan, 2008b; Hagena and Manahan-Vaughan, 2011) and enhances neuronal IEG expression in the dorsal distal CA1 and proximal CA3 regions (Hoang et al., 2018; Hoang and Manahan-Vaughan, 2024, 2025). Little is known about the extent to which changes in synaptic efficacy and neuronal encoding are triggered along the dorsoventral axis of the hippocampus by spatial experiences of this kind. However, learning about the spatial location of discretely located items, or of large LM objects that can be used as directional cues, enhance and prolong short term synaptic plasticity in the intermediate DG (Kenney and Manahan-Vaughan, 2013), and it has been reported that ventral CA1 region encodes individual stimuli features, rather than positive or negative valence of related experience (Biane et al., 2025).

Thus far it remains unclear, if and how specific components of spatial experience, particularly related to ‘what’ and ‘where’ information are processed differently in subfields of the intermediate and ventral hippocampus, and whether information encoding patterns differ from dorsal hippocampal regions. This study therefore examined how multisensory exploration of a novel spatial configuration of spatial content information, related to discrete visuospatial content information (microscale), or directional (LM, macroscale) cues, affects neuronal activity in the dorsal, intermediate, and ventral hippocampi of rats. For this, we used experience-dependent identification of nuclear expression of the immediate early gene (IEG), Homer1a, in hippocampal neurons, using fluorescence *in situ* hybridization (FISH) to tag neurons that were directly engaged by the experience (Hoang et al., 2018). Nuclear Homer1a expression is triggered in the hippocampus both by spatial learning and synaptic plasticity events, and is expressed differently along the proximodistal axis of the dorsal hippocampus, depending on whether the spatial constellations of microscale or microscale cues are being learned or updated (Hoang et al., 2018, 2021; Hoang and Manahan-Vaughan, 2025). We report here that a distinct and differentiated pattern of neuronal IEG expression occurs both the DG, as well as the proximodistal components of the cornu ammonis, of the hippocampal dorsoventral axis, as a result of microscale and macroscale spatial experience.

## 2 Materials and methods

The study was carried out in compliance with the European Communities Council Directive of September 22nd 2010 (2010/63 / EEC) for care of laboratory animals. It was approved beforehand and conducted under license of the animal ethics committee of the local government authority (Landesamt für Verbraucherschutz und Ernährung (LAVE), Nordrhein Westfalen). Sample sizes, outcome determination, statistical analysis strategies and experimental procedures complied with the ARRIVE guidelines 2.0. Analysis of the data was conducted in an experimenter-blind manner. Animal well-being was monitored daily and was accompanied by weekly health assessments by the veterinary clinician of Ruhr University Bochum. Humane end-points were implemented.

### 2.1 Animals

Sixteen male Wistar rats (7–9 weeks old) were used in this study. Females were not included in the study to avoid possible differences in behavior and immediate early gene expression that relate to sexual dimorphism (D’Souza et al., 1999; Perkins et al., 2017; Francesconi et al., 2020), which would, in turn, necessitate an increased sample size for subsequent statistical analysis of data. Animals were kept under standard housing conditions in vivaria (Scanbur A/S, Karlslude, Denmark), with a 12 h light/dark cycle that was maintained at a constant temperature (22 ± 2 °C) and humidity (55 ± 5%). Water and food were available *ad libitum*.

### 2.2 Behavioral experiments

**Figure 1:**
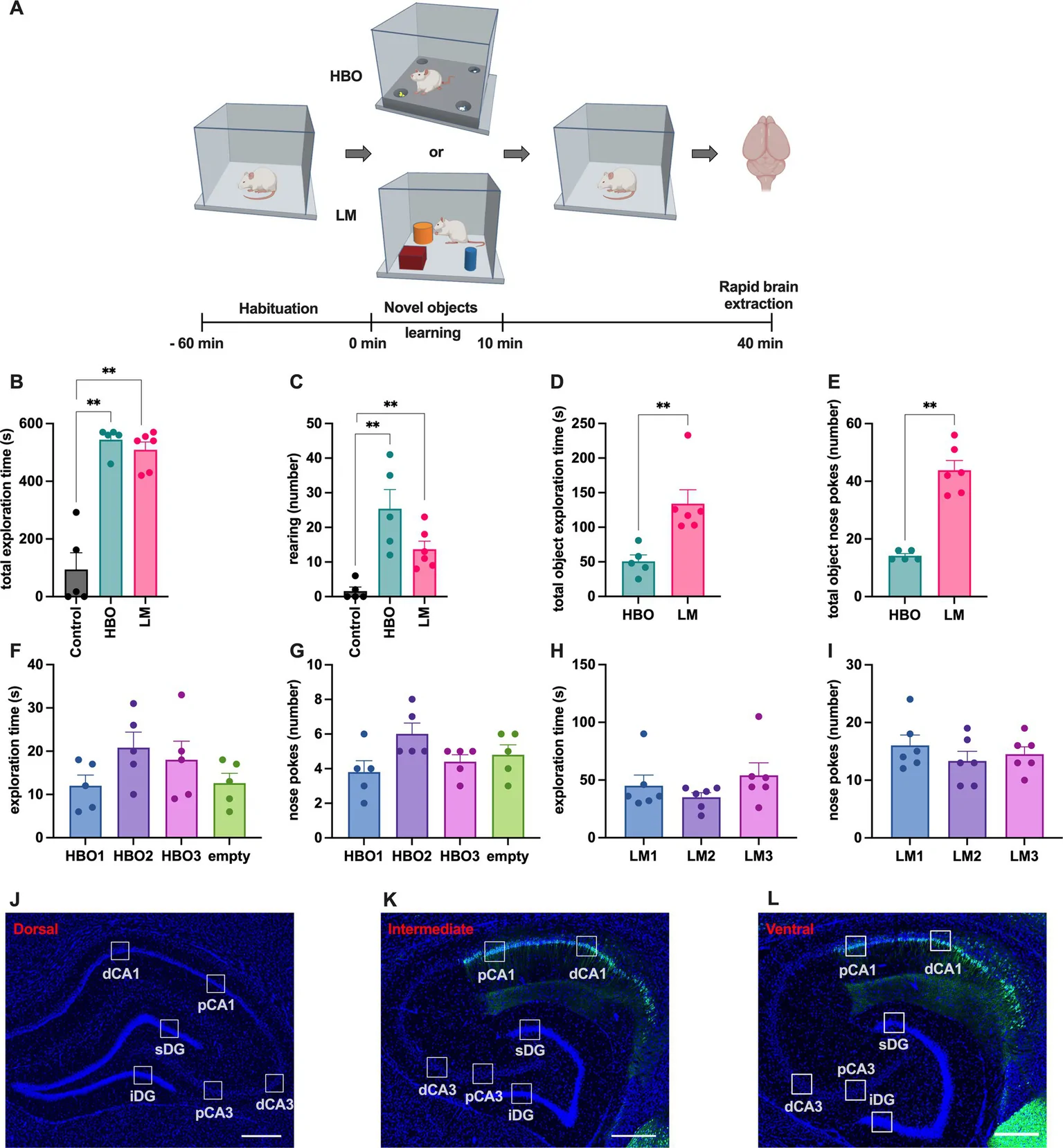
Total exploration time of holeboard or landmark environment is equivalent across animal groups. **(A)** Schema depicting the experimental design on the test day: Rats were habituated to a chamber for 1 h and were either exposed to a novel holeboard containing objects placed in the holeboard holes (HBO), or to novel landmark items (LM). Brains were extracted 40 min after the start of exploration. HBO: Three small objects were placed within three of the four holes of a holeboard. LM: Three large objects were placed on the floor of the chamber. Created using BioRender (https://BioRender.com/fjzd7n0). Exposure to either novel HBO, or to LM, animals showed a significant increase in exploration total exploration time **(B)** and rearing events **(C)** (Control vs. HBO: *U* = 0, *p* = 0.0079, Control vs. LM: *U* = 0, *p* = 0.0043 for both total exploration time and rearing events). No statistically significant differences were detected in total exploration time **(B)** and rearing events **(C)** when HBO and LM exposure were compared. **(D,E)** During HBO exploration the total object exploration time (**D**, *p* = 0.0043) and the sum of the nose pokes number of each item (**E**, *p* = 0.0043) are significantly smaller compared to LM exploration. Animals did not exhibit a significant preference for any of the four holes of the holeboard (the three holes containing objects are designated HBO1-3) or LM (items 1–3): During HBO exploration the total exploration time **(F)** as well as the number of nose pokes of each hole-board hole **(G)** were not significantly different. During LM exploration, no statistically significant differences were detected for the total exploration time **(H)** or the number of nose pokes of each LM (LM1-3) **(I)**. **(J–L)** DAPI (blue) stained images show examples of a horizontal section of the dorsal hippocampus **(J)** (~3.6 mm posterior to Bregma), a coronal section, of the intermediate hippocampus **(K)** (~5.6 mm below to Bregma), and a coronal section of the ventral hippocampus **(L)** (~7.1 mm below to Bregma). White squares represent the regions of interest, from which z-stack images were taken. WFS1 staining (green) was used to differentiate CA1 pyramidal cells from subicular cells in intermediate and ventral hippocampus **(K,L)**. dCA1, Distal CA1; dCA3, Distal CA3; pCA1, Proximal CA1; pCA3, Proximal CA3; iDG, Infrapyramidal blade of DG; sDG, Supramyramidal blade of DG. Data shown in panels **(B–I)** are presented as mean ± SEM. Individual data points represent single animals. **Mann–Whitney test, *p* < 0.01. For images shown in **(J–L)**, the scale bar corresponds to 500 μm.

Two different spatial content learning tasks were implemented. Learning about overt LM (macroscale) features of an environment promotes long-term depression (LTD; Kemp and Manahan-Vaughan, 2008b; Hagena and Manahan-Vaughan, 2011) and increases IEG expression in neuronal nuclei of the CA3 region and DG (Hoang et al., 2018). Similarly, learning about the spatial constellation of objects concealed in holeboard holes (HBO, microscale spatial learning) promotes LTD in the cornu ammonis (Kemp and Manahan-Vaughan, 2008b; Hagena and Manahan-Vaughan, 2011) and enhances IEG expression in neuronal nuclei of the CA1 and CA3 regions (Hoang et al., 2018; Hoang and Manahan-Vaughan, 2024, 2025). The same behavioral tasks were used here, as previously described for the assessments mentioned above (Hoang et al., 2018). All animals were handled by the experimenter for at least 15 min per day on two consecutive days prior to commencing apparatus habituation. Afterwards, all animals were exposed to the test chamber for 1 h per day on three consecutive days. The test chamber (40 × 40 × 50 cm) consisted of gray plastic walls with a translucent perspex door. The chamber was open at the top. Habituation and HBO/LM learning/non-learning experiments were conducted in the same recording chamber for each individual animal. On the test day, the animals were randomly divided into three groups depending on the behavioral task. They rested in the chamber for 1 h before the behavioral task was commenced. These comprised:

1. **Microscale spatial learning**: Novel exposure to a holeboard that contained novel objects in three of the holeboard holes (HBO; Figure 1A, *n* = 5). The door of the chamber was moved vertically upwards (on guiding rails) to allow the insertion of the holeboard (39.8 × 39.8 cm, gray plastic). The four holeboard holes (diameter: 5.5 cm, depth: 5 cm) were equidistant from the edges of the holeboard and from one another. Three holes contained novel small objects (ca. 4 × 2 × 2 cm). These objects comprised small toy figures, made of hard washable plastic, that differed from each other in color, appearance and size and easily fitted within the holes. The objects did not extend above the surface of the holeboard and could only be detected when the animal inserted its nose into the hole. After 1 h habituation in the test chamber, the holeboard with objects was inserted into the chamber directly without removing the animal. The holeboard covered the original floor. The animal was exposed to novel HBO for 10 min. Afterwards, the HBO was removed, and the animal resided in the chamber for another 30 min before brain removal was conducted.
2. **Macroscale spatial learning**: Novel exposure to overt cues in the form of large objects (max 11 × 10 × 10 cm) that served as landmarks (LM; Figure 1A, *n* = 6). After 1 h habituation in the test chamber, three LMs were placed into the chamber in the presence of the animal. The exposure sequences were as described for HBO.
3. **Control experiment**: Exposure to the chamber in the absence of a behavioral task (Figure 1A, *n* = 5). Here, after 1 h habituation in the test chamber, animals resided undisturbed in the chamber for the same duration of time as the test animals.

During the behavioral tasks, the animals were videotaped. The data was later analyzed offline by at least 2 trained experimenters. The experimenters were blinded to the identity (ID) of the individual animals. The timeline of the experiment was chosen to examine experience-dependent expression of the IEG Homer1a, which peaks in the nucleus 35–40 min after a specific experience, thereby making it a useful biomarker for the mapping of activity-dependent neuronal information encoding (Bottai et al., 2002).

Each brain was rapidly removed after transferring the animal gently from the test chamber to a guillotine in a continuous movement that lasted no longer than 3 s. Each brain was separated into two hemispheres. Immediately afterwards, brains were shock-frozen in 2-methylbutane (contained in a small metal vessel maintained at ca. −60 °C), surrounded by liquid nitrogen, and subsequently stored at −80 °C until further processing.

### 2.3 Fluorescence *in situ* hybridization

Brains were sliced under RNase-free conditions using a cryostat (Leica CM3050 S; Leica Biosystems GmbH, Wetzlar, Germany). The left hemisphere was sectioned in the horizontal plane, and the right hemisphere was cut in the coronal plane. This was done to accurately identify the dorsal, intermediate and ventral parts of the hippocampus. Horizontal and coronal sections (20 μm thick) were collected, mounted directly onto glass slides (Superfrost Plus, Gerhard Menzel GmbH, J1800AMNZ, Braunschweig, Germany), and stored at −80 °C until further processing.

Fluorescence in situ hybridization (FISH) was used to detect nuclear neuronal Homer1a expression that was triggered by the above mentioned learning events, and followed a previously established protocol (Hoang et al., 2018). Digoxigenin-labeled probes were created in the following manner: a Homer1a cDNA plasmid was commercially synthesized (Genscript Biotech, Piscataway, NJ, USA) based on a ~ 1.2 kb Homer1a transcript (Brakeman et al., 1997). Antisense cRNA probes were transcribed from linearized cDNA using a transcription kit (Ambion MaxiScript, Invitrogen, Thermo Fisher Scientific, Waltham, MA, USA) and a premixed RNA labeling nucleotide mixture containing digoxigenin-11-UTP (Roche Diagnostics, Basel, Switzerland). The resulting RNA probes were purified using microcentrifuge-compatible chromatography columns (Monarch Spin RNA Cleanup Kit, T2040L, New England Biolabs, Frankfurt, Germany), and probe yield and integrity were assessed by gel electrophoresis, while RNA concentration was determined using a fluorescent RNA-binding dye (QuantiFluor RNA system, Promega, Madison, WI, USA).

From each animal, coronal sections containing the dorsal hippocampal CA (~3.6 mm posterior to Bregma) and horizontal sections containing the intermediate (5.3–5.8 mm ventral to Bregma) and ventral hippocampus (6.8–7.6 mm ventral to Bregma) were collected. From each region, one slide containing 3 consecutive sections was selected and left at room temperature (RT) to thaw (~1 h). Next, slices were fixed in iced-cold 4% paraformaldehyde in phosphate buffered saline (PBS; fresh filtered) for 20 min, washed twice for 2 min each in 2 × saline-sodium citrate (SSC) buffer. The sections were then incubated in acetic anhydride solution for 10 min, rapidly washed four times for 1 min each in 2 × SSC, followed by a final 5 min wash in 2 × SSC. Then, the slides underwent a prehybridization step for 20 min in a mixture of 4 × SSC and formamide (Sigma-Aldrich, 47671, St. Louis, USA, 1:1, RNase-free) at 37 °C. The labeled RNA probes were diluted to a final concentration of 1 ng/μL in 1 × hybridization buffer (Sigma-Aldrich, H7782, St. Louis, USA), heated at 90 °C for 5 min, and then rapidly cooled on ice. A humid chamber (RNase-free) was prepared with a mixture of 4 × SSC and formamide soaked filter paper. Sections were incubated in hybridization buffer containing labeled RNA probes within the humid chamber, covered with self-sealing thermoplastic (Parafilm, Bemis, PM996, Neenah, WI, USA) and a glass coverslip to prevent evaporation. Sections were then hybridized for ~17 h at 56 °C. A ‘negative control’ slide, containing hybridization buffer *only,* was included in parallel. Following hybridization, slides underwent stringent washing steps: thrice in 2 × SSC at 56 °C (5 min each), once in 2 × SSC containing RNase A at 37 °C (15 min), once in 2 × SSC at 37 °C (10 min), twice in 0.5 × SSC at 56 °C (10 and 30 min), once in 0.5 × SSC at RT (10 min), twice in 1 × SSC at RT (5 min each), and finally thrice in tris-buffered saline (TBS) at RT (5 min each).

Signal detection was performed using immunohistochemistry. Slides were treated with 3% H₂O₂ at RT for 15 min to block endogenous peroxidase activity, washed four times in TBS (5 min each), incubated in blocking solution (Leica, PV6122, Leica Biosystems GmbH, Heidelberger, Germany) for 70 min, and then incubated for 90 min with anti-digoxigenin peroxidase (1:2000, Roche Holding AG, 11207733910, Basel, Switzerland) in blocking solution, followed by rinsing four times in TBS (5 min each). Slides were subsequently incubated for 20 min in TBS containing 0.5% biotinylated tyramide and 0.005% H₂O₂, washed four times in TBS (5 min each), incubated for 60 min in TBS-Tween 0.05% containing 1% normal goat serum (Histoprime, ENG9010, Biozol, Hamburg, Germany) and streptavidin-Cy5 (Jackson Immuno Research, 016–170-048, Scottsdale, Arizona, USA), and finally washed four times in TBS (5 min each).

### 2.4 Immunohistochemistry

To better delineate CA1 pyramidal cells, particularly in intermediate and ventral hippocampal regions where they partially overlap with cells of subiculum, Wolfram syndrome 1 protein (WFS1) expression was conducted (Takeda, 2001). This was done by immunohistochemical tissue processing after the FISH procedure (Sethumadhavan et al., 2022). Slides were treated with 3% H₂O₂ at RT for 15 min to block endogenous peroxidase activity, washed four times in TBS (5 min each), blocked for 90 min in TBS-Tx 0.2% containing 10% normal goat serum and 20% streptavidin (Vector Laboratories, SP-2002, Burlingame, CA, USA), and then incubated overnight at 4 °C in TBS-Tx 0.2% containing 1% normal goat serum, 20% biotin (Vector Laboratories, SP-2002, Burlingame, CA, USA), and WFS1 rabbit polyclonal antibody (1:500; Proteintech, 26995-1-AP, Rosemont, IL, USA). Slides were washed four times in TBS (5 min each), incubated for 90 min with TBS-Tx 0.1% containing 1% normal goat serum and goat anti-rabbit-antibody conjugated to Cy2 (1:500, Jackson Immuno Research, 111–225-003, Scottsdale, Arizona, USA), and washed four times in TBS (5 min each).

Following WFS1 staining, slides were rinsed in double-distilled water, briefly dipped in 70% ethanol, and then stained for 10 min with 1% Sudan Black B (Merck KGaA, 199,664, Sigma-Aldrich, St. Louis, MO, USA) in 70% ethanol (Oliveira et al., 2010). Slides were subsequently rinsed in double-distilled water, air-dried, and mounted in antifade mounting medium (immunoSelect®, SCR-038447, Dianova, Biozol, Hamburg, Germany) containing 4′,6-diamidino-2-phenylindole (DAPI).

### 2.5 Data processing and statistical analysis

Visualization and statistical analyses were performed using GraphPad Prism 10 (GraphPad Software, LLC Boston, MA, USA).

#### 2.5.1 Behavioral tasks

The total exploration time during the 10-min novel HBO/LM exposure was calculated as the time the animal spent exploring in the chamber, i.e., the duration of all behaviors except resting, sleeping, and grooming. Rearing was defined as an event in which the animal freely rose onto its hind paws, without forepaw contact with the chamber wall or LM items.

HBO exploration was quantified as follows: for each object, the number of nose pokes was calculated based on the number of times a rat dipped its nose inside a holeboard hole (independent of how long these dips lasted). The object exploration time was defined as the total time the nose of the rat was inside a hole that contained an object: HBO1-3 describes exploration of a hole that contained an object. Nose pokes into the empty holeboard hole were also assessed. To assess LM exploration, the number of nose pokes was defined as the number of times the rat oriented its nose toward the object at a distance of less than 1 cm, or contacted the object for at least 2 s, and the object exploration time was defined as the total time the rat explored the object (within these criteria). The total nose poke number, and exploration time were calculated as sum of the measures for all objects (excluding the empty hole for HBO exploration).

Normal distribution of the behavioral data was tested using the Shapiro–Wilk test. Some datasets were not normally distributed, therefore behavioral data were analyzed using Mann–Whitney or Kruskal-Wallis tests. Data are presented as mean ± SEM. Statistical significance was set at *p* < 0.05.

#### 2.5.2 IEG expression

Regions of interest (ROIs) containing distal CA1 (dCA1), proximal CA1 (pCA1), distal CA3 (dCA3), proximal CA3 (pCA3) regions, suprapyramidal blade of DG (sDG) and infrapyramidal blade of DG (iDG; Figures 1J–L) were annotated using z-stack images acquired at 63 × magnification with a wide-field fluorescence microscope (ApoTome, Carl Zeiss AG, Oberkochen, Germany). Each ROI comprised a standardized area of 150 × 200 μm. All analyses were performed in an experimenter-blind manner. For all DG, CA1 and dorsal CA3 regions, DAPI-labeled nuclei were manually quantified using an open-source image-processing tool (Fiji Image-J,^1^ Schindelin et al., 2012), and Homer1a positive (Homer1a+) nuclei were identified to calculate the percentage of positive cells. In intermediate and ventral CA1 regions, only WFS1-positive cells were analyzed. For intermediate and ventral CA3 regions (characterized by sparse cell density), z-stack images were merged via orthogonal projections, imported into open source software for digital whole slide image analysis (QuPath,^2^ automatically segmented using open-source deep learning based nuclei/cell detection and segmentation method (StarDist,^3^ Schmidt et al., 2018) for DAPI nuclei (with further manual verification). Homer1a-positive nuclei were also manually identified to calculate the percentage of positive cells. Three consecutive sections per animal were analyzed, and means were calculated. Then the average of each group was calculated.

Data are presented as mean ± SEM. Normality of data was first assessed with the Shapiro–Wilk test. All data were normally distributed, therefore a two-way analysis of variance (ANOVA), followed by Fisher’s LSD post-hoc tests, were used for statistical analysis. Data are presented as mean ± SEM. Statistical significance was set at *p* < 0.05.

## 3 Results

### 3.1 Increasing the spatial content of a known environment is associated with significant item-specific exploration

In order to assess nuclear IEG expression that is triggered by spatial content learning, we first verified that altering the content of a spatial environment results in increased feature exploration. When exposed to either novel items in a novel holeboard (HBO), or to large overt novel items (LM), animals showed a significant increase in exploration that was specifically focused on the spatial content changes (Mann–Whitney test: total exploration time: Control vs. HBO: U = 0, *p* = 0.0079, Control vs. LM: U = 0, *p* = 0.0043, rearing number: Control vs. HBO: U = 0, *p* < 0.01, Control vs. LM: U = 0, *p* < 0.01, Figures 1B,C). During the 10 min exposure period, the total exploration time (U = 7, *p* = 0.1753, Figure 1B), as well as the number of individual rears (U = 5.500, *p* = 0.0909, Figure 1C), did not differ between HBO and LM groups. A comparison of the summed exploration duration and nose poke numbers for all objects (total object exploration time and counts) in each group revealed that both the total number of object-only nose pokes explorations (U = 0, *p* < 0.01) and the total object exploration time (U = 0, *p* < 0.01) were significantly lower in the HBO group compared to the LM group. This may be due to the increased effort involved in approaching the holeboard holes and examining their contents (Figures 1D,E). Animals showed no preference for any of the four holeboard holes (Figures 1F,G) or the three LM objects (LM1-3, Figures 1H,I): No significant differences were observed in exploration time of the holeboard holes that contained objects (HBO 1–3) or the empty hole (Kruskal-Wallis test, H(3) = 4.639, *p* = 0.2002). The number of nose pokes into each holeboard hole was also equivalent (H(3) = 5.485, *p* = 0.1395; Figures 1F,G). Similarly, in the LM group, there were no significant differences in the duration of exploration (H(2) = 3.531, *p* = 0.1754), or the nose poke number, with regard to each LM object (H(2) = 0.9375, *p* = 0.6488; Figures 1H,I).

### 3.2 Novel microscale learning increases nuclear Homer1a expression in dorsal and intermediate distal CA1, whereas macroscale learning results in increased nuclear Homer1a expression in ventral distal CA1 and proximal CA1 regions

As a next step, nuclear Homer1a mRNA expression in neurons of the cornu ammonis was examined. Here, the subfields were differentiated into distal and proximal parts of CA1 and CA3, and expression levels along the dorsoventral hippocampal axis was examined.

**Figure 2:**
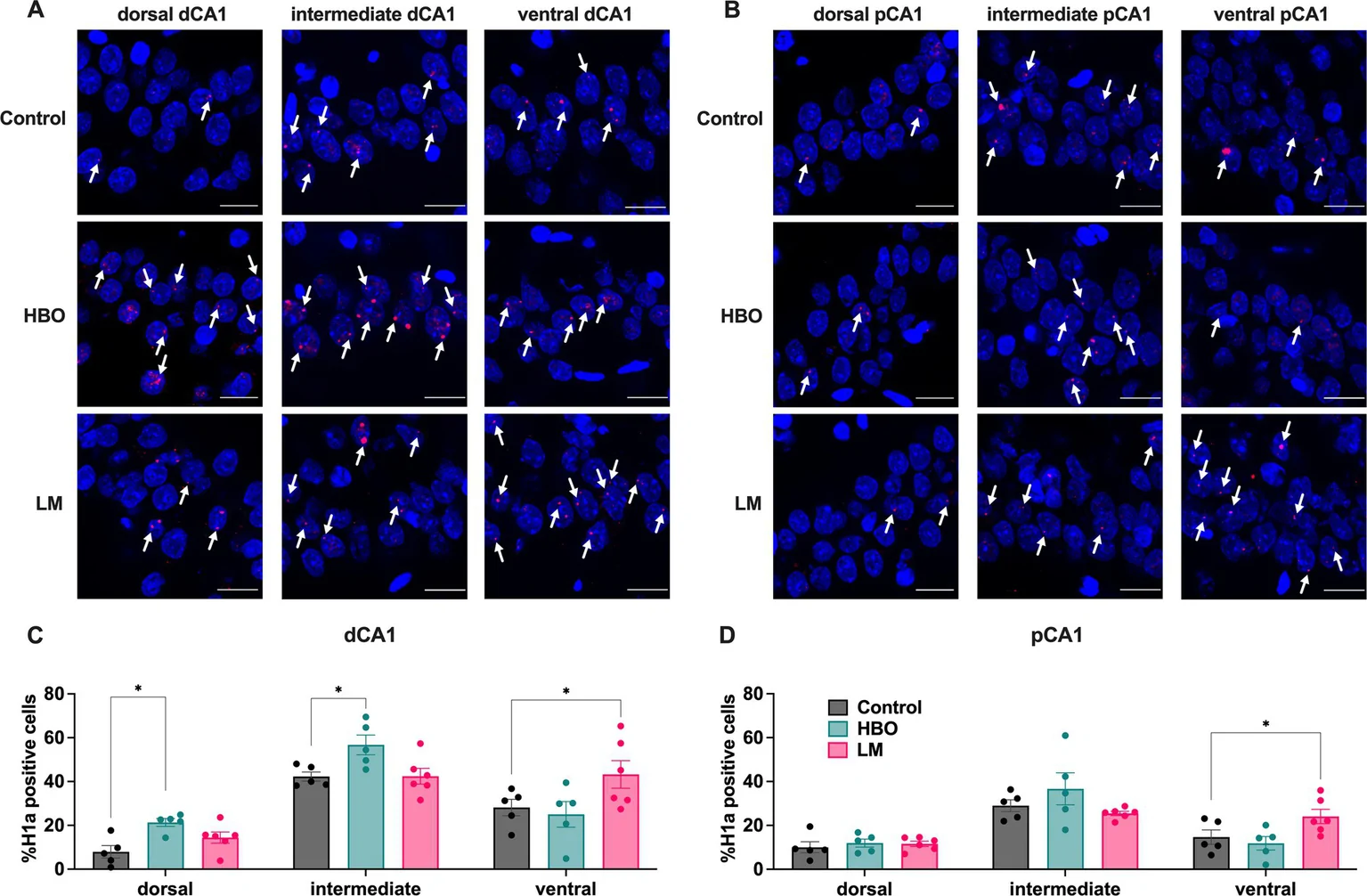
Novel microscale spatial learning results in an increase in nuclear Homer1a expression in dorsal and intermediate dCA1 region. By contrast, novel macroscale learning increases nuclear Homer1a expression in ventral dCA1 and pCA1 regions. **(A,B)** Representative images of Homer1a (H1a)-positive nuclei (red, indicated with white arrows) in **(A)** distal CA1 (dCA1) and **(B)** proximal CA1 (pCA1) region across dorsal, intermediate, and ventral hippocampal divisions in control, microscale (HBO) and macroscale (LM) groups. Nuclei are stained with DAPI (blue). Five layers of z-stack images (interval 0.7 μm) were merged for visualization. Scale bar: 20 μm. **(C)** Novel HBO learning results in a significant increase in H1a-positive nuclei in dorsal (*p* = 0.0185) and intermediate dCA1 (*p* = 0.0117) regions in comparison to control animals. In contrast, in the ventral dCA1 region, nuclear H1a expression is increased by novel LM learning (*p* = 0.0119). **(D)** H1a expression in the ventral pCA1 region is significantly increased by LM learning compared to the control group (*p* = 0.0117). **(C,D)** Data are presented as mean ± SEM. Individual data points represent single animals. Statistical significance is indicated as two-way ANOVA followed by Fisher’s LSD *p < 0.05.

Nuclear Homer1a expression in the distal CA1(dCA1; Figure 2C) significantly differed between the HBO and control groups, and was also significantly different along the dorsoventral axis (two-way ANOVA: group effect, F(1,24) = 7.258, *p* < 0.05; dorsoventral axis effect, F(2,24) = 44.79, *p* < 0.0001; interaction, F(2,24) = 3.463, *p* < 0.05). Outcomes of post-hoc Fisher’s LSD tests are summarized in Table 1. Consistent with previous reports (Hoang et al., 2018; Hoang and Manahan-Vaughan, 2024, 2025), ‘microscale’ learning of a spatial configuration of three discrete objects in three of four holes of a holeboard significantly increased nuclear Homer1a mRNA expression in neurons of the dorsal dCA1 compared to control animals (Figure 2C; Table 1). In addition, the intermediate dCA1 revealed significantly higher Homer1a expression triggered by novel HBO (microscale) spatial learning, than was detected in controls, whereas the ventral dCA1 exhibited no changes (Figure 2C; Table 1).

**Table 1:** Summary of statistical results (*post-hoc* test) evaluating changes in nuclear Homer1a expression in hippocampual neurons by novel microscale (HBO) /macroscale (LM) spatial learning compared to control animals.

Dorsoventral axis	Group	dCA1	pCA1	dCA3	pCA3	sDG	iDG
		*p* value	*p* value	*p* value	*p* value	*p* value	*p* value
dHPC	Control vs. HBO	**<0.05**	0.7253	0.5301	**<0.01**	0.8029	0.6812
	Control vs. LM	0.2553	0.6554	0.3653	**<0.0001**	0.3300	**<0.0001**
iHPC	Control vs. HBO	**<0.05**	0.1746	0.3914	0.3666	0.1180	0.3939
	Control vs. LM	0.9781	0.3155	0.2724	0.9729	0.6902	0.3799
vHPC	Control vs. HBO	0.5606	0.6098	0.2665	0.9090	0.5201	0.7724
	Control vs. LM	**<0.05**	**<0.05**	0.9364	**<0.05**	0.28157	0.2661

The table reports the post hoc statistical outcomes, obtained using Fisher’s LSD test after two-way ANOVA comparing nuclear Homer1a expression detected in HBO or LM groups with expression in the control group. *p* values for comparisons between controls and HBO or LM groups are presented. Statistically significant differences (*p* < 0.05) are highlighted in bold. dHPC, dorsal hippocampus; iHPC, intermediate hippocampus; vHPC, ventral hippocampus; HBO, holeboard, containing objects; LM, landmarks; dCA1, distal CA1; pCA1, proximal CA1; dCA3, distal CA3; pCA3, proximal CA3; iDG, infrapyramidal blade of dentate gyrus; sDG, suprapyramidal blade of dentate gyrus.

Similarly, nuclear Homer1a expression in neurons of dCA1 (Figure 2C) significantly differed between the LM and control groups, and was also significantly different along the dorsoventral axis (two-way ANOVA: group effect, F(1,27) = 5.040, *p* < 0.05; dorsoventral axis effect, F(2,27) = 34.45, *p* < 0.0001; interaction, F(2,27) = 1.796, *p* = 0.1852). Post-hoc Fisher’s LSD tests revealed that ‘macroscale’ learning of LM objects had no effect on Homer1a expression in the dorsal or intermediate dCA1, but significantly increased Homer1a expression in the ventral dCA1 in comparison with control animals (Figure 2C; Table 1).

With regard to the proximal CA1(pCA1), Homer1a expression was also only significantly different along the dorsoventral axis (two-way ANOVA HBO vs. control: group effect, F(1,24) = 0.5106, *p* = 0.4818; dorsoventral axis effect, F(2,24) = 19.18, *p* < 0.0001; interaction, F(2,24) = 0.9205, *p* = 0.4119); two-way ANOVA LM vs. control: group effect, F(1,27) = 1.518, *p* = 0.2286; dorsoventral axis effect, F(2,27) = 22.47, *p* < 0.0001; interaction, F(2,27) = 3.526, *p* < 0.05). Post-hoc Fisher’s LSD tests revealed no differences in Homer1a expression in HBO and LM groups in the dorsal and intermediate pCA1 region compared to controls, whereas in the ventral pCA1 the LM group exhibited significantly higher Homer1a expression compared to control animals (Figure 2D; Table 1).

In brief, context-dependent IEG expression in dorsal and intermediate CA1 is similar, whereby novel macroscale (LM) spatial learning results in no change in nuclear Homer1a expression in neurons of dCA1 or pCA1, and novel microscale (HBO) spatial learning elicits significant increases only in dCA1. By contrast, the ventral hippocampus did not exhibit any change in Homer1a expression in response to novel microscale spatial learning exposure, but showed significant increases in Homer1a expression in both dCA1 and pCA1 as a result of novel macroscale (LM) spatial learning.

### 3.3 Both novel microscale and macroscale spatial learning result in differentiated increases in Homer1a expression along the dorsoventral axis of the proximal CA3 region, with no detectable changes occuring in the distal CA3 region

It has been previously reported that commissural-associational (AC) synapses of the dorsal CA3 region respond to novel microscale (HBO) spatial learning with the expression of LTD, whereas the mossy fiber (MF)-CA3 synapses respond to novel *macroscale* (LM) spatial learning with LTD (Hagena and Manahan-Vaughan, 2011). Whereas dCA3 receives more AC (versus MF) inputs than pCA3, by contrast, pCA3 receives more MF (versus AC) inputs than dCA3 (Sun et al., 2017; Lee et al., 2020). Here, we examined responses in the distal CA3 (dCA3) and proximal CA3 (pCA3) regions of the dorsoventral axis under HBO and LM learning conditions.

**Figure 3:**
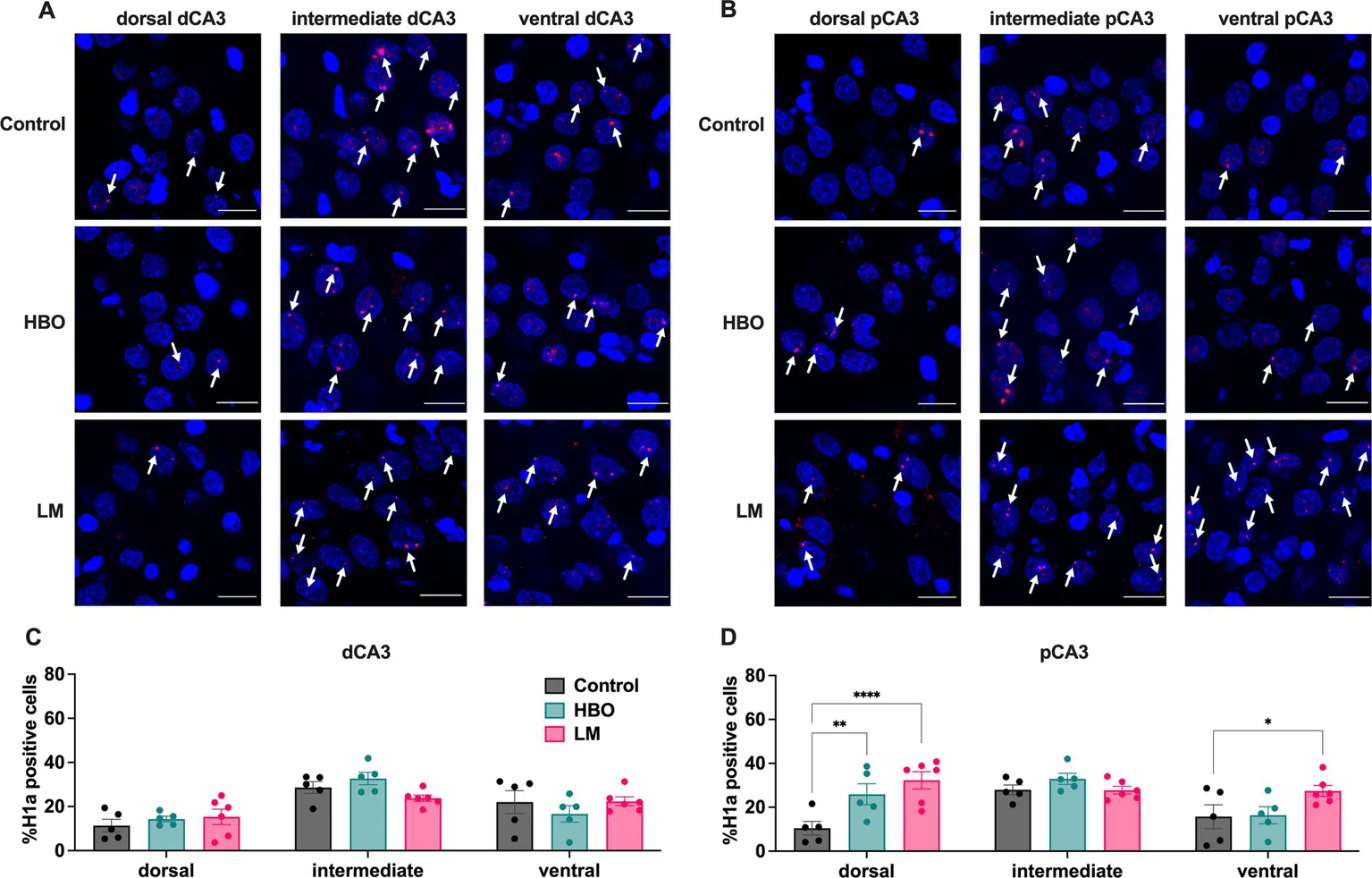
Both novel microscale and macroscale spatial learning trigger nuclear Homer1a expression in dorsal pCA3 region, while novel macroscale learning also increases nuclear Homer1a expression in ventral pCA3 region. **(A,B)** Representative images of Homer1a (H1a)-positive nuclei (red, indicated with white arrows) in **(A)** distal CA3 (dCA3) and **(B)** proximal CA3 (pCA3) region across dorsal, intermediate, and ventral hippocampal divisions of control, microscale (HBO), and macroscale (LM) groups. Nuclei are stained with DAPI (blue). Five layers of z-stack images (interval 0.7 μm) were merged for visualization. Scale bar: 20 μm. **(C)** In dorsal, intermediate, and ventral dCA3 regions, nuclear H1a expression triggered by novel HBO and LM learning is not significantly different compared to control animals. **(D)** Novel microscale (HBO) and macroscale (LM) learning result in a significant increase in nuclear H1a expression in the dorsal pCA3 region compared to control animals (HBO: *p* = 0.0086, LM: *p* < 0.0001). In the ventral pCA3 region, a significant increase in H1a expression is triggered by LM learning (*p* = 0.0192). **(C,D)** Data are presented as mean ± SEM. Individual data points represent single animals. Statistical significance is indicated as two-way ANOVA followed by Fisher’s LSD *p < 0.05, **p < 0.01, ****p < 0.0001.

Nuclear Homer1a expression in neurons of the dCA3 region (Figure 3C) did not differ between the groups, but nonetheless, significantly differed in a context-independent manner along the dorsoventral axis (two-way ANOVA HBO vs. control: group effect, F(1,24) = 0.04622, *p* = 0.8316; dorsoventral axis effect, F(2,24) = 14.81, *p* < 0.0001; interaction, F(2,24) = 1.208, *p* = 0.3164); two-way ANOVA LM vs. control: group effect, F(1,27) = 0.004726, *p* = 0.9457; dorsoventral axis effect, F(2,27) = 9.102, *p* = 0.0010; interaction, F(2,27) = 1.053, *p* = 0.3629).

By contrast, in the pCA3 region, neuronal nuclear Homer1a expression significantly differed between the HBO and control group, and also differed along the dorsoventral axis (two-way ANOVA: group effect, F(1,24) = 5.068, *p* < 0.05; dorsoventral axis effect, F(2,24) = 8.240, *p* < 0.01; interaction, F(2,24) = 1.996, *p* = 0.1578). For LM and control groups, nuclear Homer1a expression only significantly differed between the group factor (two-way ANOVA: group effect, F(1,27) = 16.85, *p* < 0.001; dorsoventral axis effect, F(2,27) = 2.478, *p* = 0.1028; interaction, F(2,27) = 5.502, *p* < 0.01). Outcomes of post-hoc Fisher’s LSD tests are summarized in Table 1. Consistent with previous findings, both novel HBO and novel LM spatial learning significantly enhanced nuclear Homer1a expression in neurons of the dorsal pCA3 region, in comparison with control animals (Figure 3D; Table 1). No differences in nuclear Homer1a expression were detectable for the HBO or LM groups, in comparison with control responses in the intermediate pCA3. However, the ventral pCA3 region showed significantly increased Homer1a expression in response to novel LM exposure (Figure 3D; Table 1). By contrast, the ventral pCA3 region did not exhibit changes in Homer1a expression in response to novel microscale learning (Figure 3D; Table 1).

### 3.4 Novel macroscale learning increases nuclear Homer1a expression only in neurons of the infrapyramidal blade of the dorsal dentate gyrus, whereas microscale learning does not elicit any response change in the dentate gyrus

**Figure 4:**
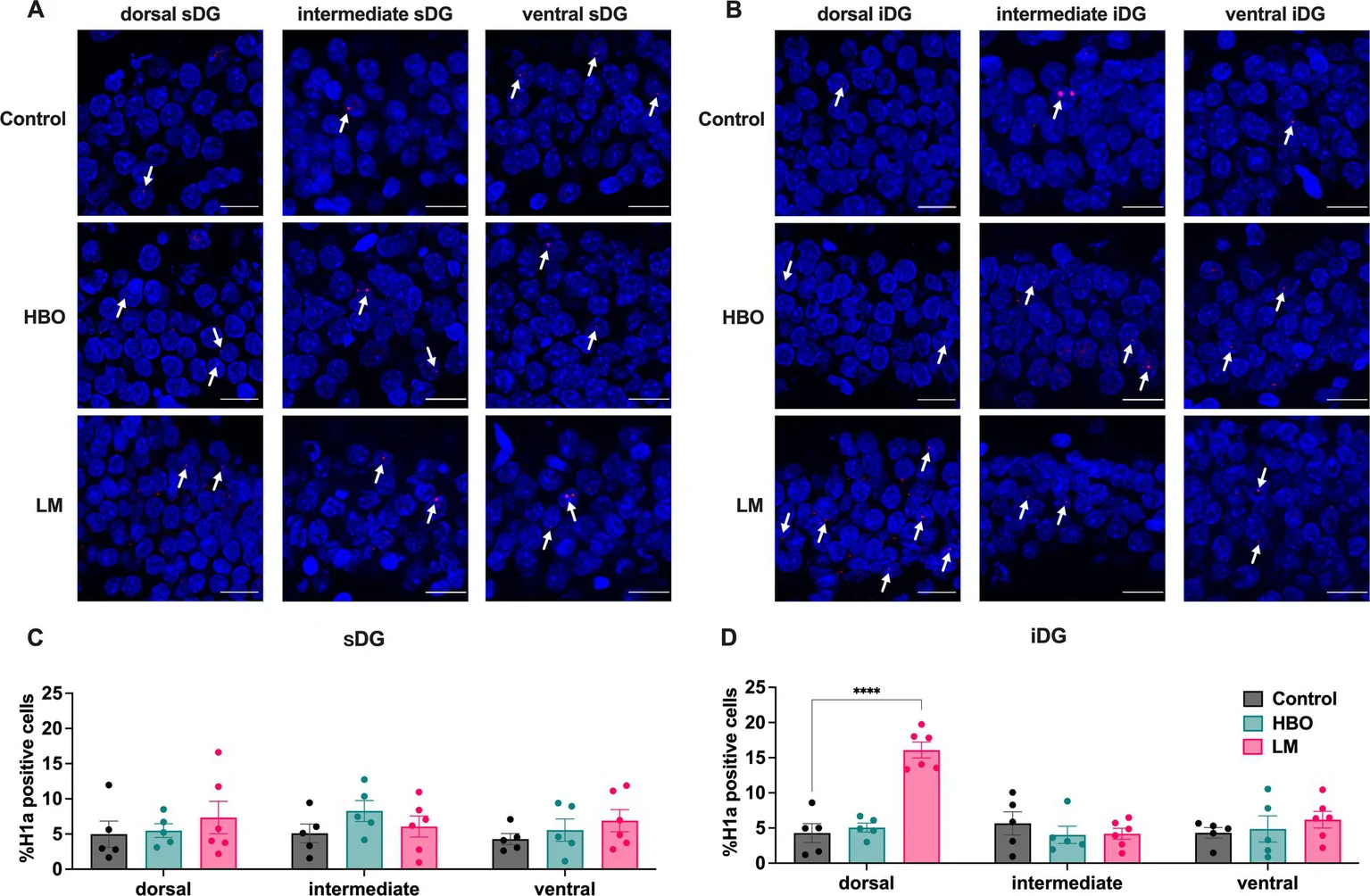
Nuclear Homer1a expression in the dorsal iDG is only increased by novel macroscale spatial learning. **(A,B)** Representative images of Homer1a (H1a)-positive nuclei (red, indicated with white arrows) in **(A)** the suprapyramidal blade of DG (sDG) and **(B)** the infrapyramidal blade of DG (iDG) across the dorsal, intermediate, and ventral hippocampal subdivisions in control, microscale (HBO), and macroscale (LM) learning groups. Nuclei are stained with DAPI (blue). Five layers of z-stack images (interval 0.7 μm) were merged for visualization. Scale bar: 20 μm. **(C)** In dorsal, intermediate, and ventral sDG, nuclear H1a expression does not differ as a result of HBO or LM learning, compared to control animals. **(D)** Novel LM spatial learning results in a significant increase in H1a expression in the dorsal iDG compared to control animals (*p* < 0.0001), while expression in intermediate and ventral iDG remains unchanged. **(C,D)** Data are presented as mean ± SEM. Individual data points represent single animals. Statistical significance is indicated as two-way ANOVA followed by Fisher’s LSD ****p < 0.0001.

Nuclear Homer1a expression in neurons of the iDG (Figure 4D) differed significantly between the LM and control groups, and also differed along the dorsoventral axis (two-way ANOVA: group effect, F(1,27) = 18.21, *p* < 0.001; dorsoventral axis effect, F(2,27) = 12.78, *p* = 0.0001; interaction, F(2,27) = 17.48, *p* < 0.0001). Post-hoc Fisher’s LSD tests revealed a significant increase of Homer1a expression only in the dorsal iDG following LM learning, but not in other parts of the DG (Figure 4D; Table 1). By contrast, novel HBO exposure failed to trigger significant changes in nuclear Homer1a expression in the iDG compared to the controls, regardless of the dorsal-ventral parts scrutinized [Figure 4D, two-way ANOVA: group effect, F(1,24) = 0.008500, *p* = 0.9273; dorsoventral axis effect, F(2,24) = 0.01930, *p* = 0.9809; interaction, F(2,24) = 0.5018, *p* = 0.6116].

Moreover, no significant differences were detected in nuclear Homer1a expression in neurons of the sDG between groups, or along the dorsoventral axis [Figure 4C, two-way ANOVA HBO vs. control: group effect, F(1,24) = 2.127, *p* = 0.1576; dorsoventral axis effect, F(2,24) = 0.9249, *p* = 0.4103; interaction, F(2,24) = 0.4954, *p* = 0.6154]; two-way ANOVA LM vs. control: group effect, F(1,27) = 2.073, *p* = 0.1614; dorsoventral axis effect, F(2,27) = 0.08012, *p* = 0.9232; interaction, F(2,27) = 0.1405, *p* = 0.8696). This finding suggests that the sDG is not involved in encoding of this kind of spatial content information.

Consistent with previous findings (Hoang et al., 2018), novel macroscale (LM) spatial learning significantly increased nuclear Homer1a expression in neurons of the dorsal iDG, but not sDG, compared to controls (Figure 4D). No differences in Homer1a expression were detectable in the intermediate or ventral DG of the HBO or LM groups, compared to controls (Figure 4D). Overall, these findings indicate that the two behavioral manipulations not only evoked different exploratory behaviors, but also engaged distinct patterns of hippocampal activation within both the proximodistal and dorsoventral hippocampal axes (Supplementary Table 1).

## 4 Discussion

**Figure 5:**
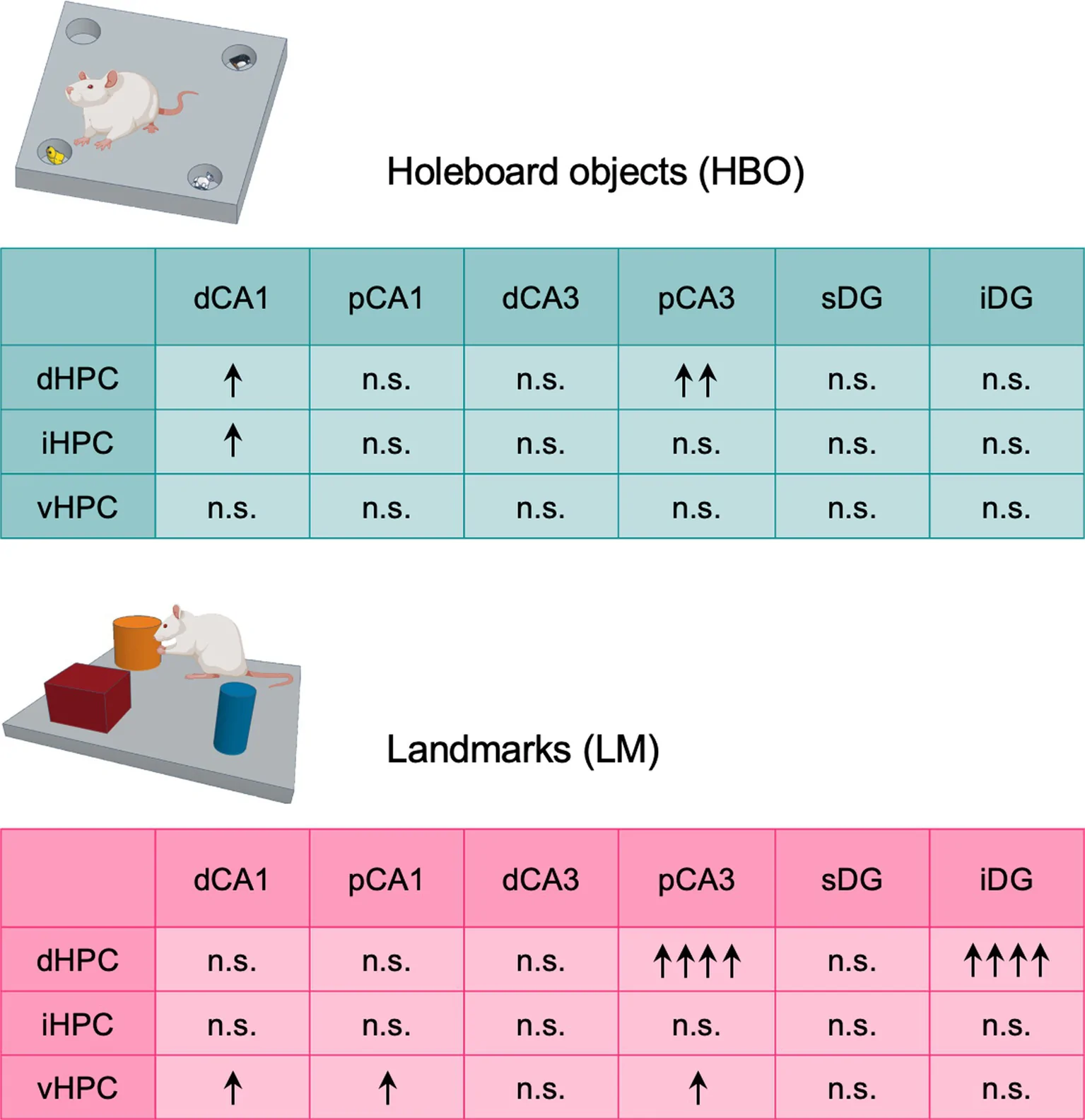
Comparisons of nuclear Homer1a expression in hippocampal neurons, as detected in animals exposed to novel HBO or LM compared to controls. The figure summarizes all pairwise statistical comparisons between HBO/LM group to the control group corresponding to the data presented in Figures 2–4. Symbols represent significance as determined by Fisher’s LSD tests following two-way ANOVA: N.s. indicate no significant effect; ‘↑’ indicating *p* < 0.05, ‘↑↑‘indicating *p* < 0.01, ‘↑↑↑’ indicating *p* < 0.001, ‘↑↑↑↑’ indicating *p* < 0.0001. dHPC, Dorsal hippocampus; iHPC, Intermediate hippocampus; vHPC, Ventral hippocampus; HBO, Holeboard containing objects; LM, Landmarks; dCA1, Distal CA1; pCA1, Proximal CA1; dCA3, Distal CA3; pCA3, Proximal CA3; sDG, Suprapyramidal blade of dentate gyrus; iDG, Infrapyramidal blade of dentate gyrus.

Subfield-specific encoding of different components of spatial experience has been reported for the proximodistal dorsal hippocampus (Chawla et al., 2005; Sauvage et al., 2013; Hagena and Manahan-Vaughan, 2024). Here, we explored whether the same kind of functional segregation is evident along the dorsoventral hippocampus, as well as the extent to which the ventral hippocampus engages in spatial information encoding. Our findings indicate that although the dorsal hippocampus is the dominant area for the encoding of spatial content information, similarities, especially at the level of encoding discrete features of spatial content, are shared with the intermediate hippocampus (Figure 5).

We used FISH to identify neurons of the hippocampus that engaged in nuclear expression of Homer1a, as a result of a novel spatial content learning experience. We observed that both the dorsal pole of the hippocampus as well as the intermediate region, exhibit increased nuclear IEG expression in neurons of the dCA1 as a result of novel microscale visuospatial encoding. In the dorsal pole, pCA3 is also engaged by novel microscale experience. The ventral pole does not engage in this kind of spatial content learning. By contrast, the dorsal pCA3 and the infrapyramidal blade of the dorsal DG encode novel macroscale information. Here, the intermediate hippocampus is not engaged at all, neither in its CA or DG. The ventral hippocampus also processes spatial information, but subfield engagement is quite distinct: its dentate gyrus did not respond at all to this kind of spatial content learning experienced by the animals, rather dCA1, pCA1 and pCA3 of the ventral pole all exhibited increased neuronal IEG expression in response to novel macroscale visuospatial learning.

### 4.1 Relevance of the two streams hypothesis for the dorsoventral hippocampal axis

The “two streams” hypothesis originated from primate studies conducted by Mishkin et al. (1983). It describes how visual item features (“what” information) are relayed and processed via cortical structures such as the perirhinal cortex and lateral EC (LEC). By contrast, spatial context (“where” information) is processed via the postrhinal cortex and medial EC (MEC; Mishkin et al., 1983). These findings, and the two streams hypothesis, were subsequently extended, on the basis of anatomical studies, to include the proximodistal axis of the dorsal hippocampus (Amaral and Witter, 1989; Ishizuka et al., 1990; Witter et al., 2000; Fyhn et al., 2004; Hargreaves et al., 2005; Manns and Eichenbaum, 2006; Furtak et al., 2007; Witter, 2007; Henriksen et al., 2010; Burke et al., 2011; Ito and Schuman, 2012; Sauvage et al., 2013).

Behavioral studies of different aspects of associative memory have indicated the dCA1 and pCA3 regions of the dorsal hippocampus preferentially encode visual item features, whereas its pCA1 and dCA3 regions favor visuospatial information processing (Henriksen et al., 2010; Burke et al., 2011; Ito and Schuman, 2012; Sauvage et al., 2013). However, the LEC projects specifically to dCA1, whereas MEC inputs extend across both dCA1 and pCA1 (Li et al., 2017). This suggests that although dCA1 may be more specialized toward the encoding of non-spatial information, it also integrates spatial information. In the present study, we identified significant increases in nuclear IEG expression in neurons of the dorsal dCA1 and pCA3 when animals were exposed to novel constellations of items that were partially concealed in holeboard holes. We refer to this kind of spatial learning as ‘microscale’, because in order to find the objects, the animals have to approach the holes and examine their contents. Thus, they had to not only learn the location of the items, but also their identity. This would be consistent with an integration of spatial and non-spatial information mediated by MEC and LEC inputs to dCA1. Interpreted on the basis of the findings mentioned above (Li et al., 2017), it is curious that neurons of the dorsal pCA1 did not exhibit significant changes in nuclear IEG expression, suggesting that spatial information *alone* was insufficient in driving information encoding.

In line with a putatively significant role of dCA1 in the encoding of non-spatial visual (item) information within a spatial context, we found that the intermediate dCA1 also showed significant IEG expression following microscale spatial learning. Unlike dorsal cornu ammonis, pCA3 of the intermediate hippocampus did not respond. This could suggest that the dorsal cornu ammonis integrates both spatial and non-spatial aspects of associative experience, while intermediate cornu ammonis favors information encoding about discrete features of spatial content. In line with this interpretation, it was reported that that CA1 and CA3 exhibit a functional differentiation in terms of spatial versus non-spatial visual information processing along the dorsoventral axis (Beer et al., 2014). Bidirectional interactions between dorsal and intermediate dCA1 (Swanson et al., 1978; Tao et al., 2021) may have supported the mutual encoding of microscale information described here. One possible explanation for the relative inertia of the intermediate hippocampus with regard to the kinds of spatial content learning assessed in the present study, could be the absence of a specific goal or valence for these tasks: The intermediate hippocampus receives denser projections from the amygdala and ventral tegmental area (VTA) than does the dorsal hippocampus (Gasbarri et al., 1994; Pikkarainen et al., 1999) and has been ascribed roles in dynamic task switching, motivated by escape from a water maze (Bast et al., 2009), and goal/value-directed navigation (Hwang et al., 2024).

The ventral cornu ammonis may support non-spatial encoding by the dorsal cornu ammonis by means of information provided by a monodirectional projection from ventral CA1 to dorsal CA3 (Lin et al., 2021). Projections from dorsal hippocampus to the ventral pole and vice versa are, however, sparse (Swanson et al., 1978; Tao et al., 2021). Here too, the relatively low activation of the ventral hippocampus by microscale and macroscale spatial learning, might be explained by the low emotional valence of the tasks. The ventral hippocampus has dense interconnectivity with the medial prefrontal cortex (Hoover and Vertes, 2007; Liu and Carter, 2018), as well as the ventral tegmental area (VTA), and exhibits reciprocal connectivity with the amygdala (Gasbarri et al., 1994; Pikkarainen et al., 1999; Felix-Ortiz and Tye, 2014). Taken together, these findings suggest that the two streams hypothesis, i.e., the processing of spatial and non-spatial visual information within the proximodistal axis of the cornu ammonis, is only relevant for the dorsal hippocampus. This is likely to subserve the precision of spatial navigation, spatial memory and the building of experience-dependent associations. The processing of spatial information by the intermediate and ventral subdivisions, may subserve the integration of valence and motivation into these representations. This possibility is supported by studies that have described a role for the intermediate hippocampus in cue-based task switching (Jarrard et al., 2012) and reward prediction (Jarzebowski et al., 2022), and of the ventral hippocampus in social memory (Okuyama et al., 2016) and motivation-based behavior (Komorowski et al., 2013; Ciocchi et al., 2015).

### 4.2 Division of labor with regard to spatial information processing along the dorsoventral axis of the hippocampus

The entirety of the dorsoventral hippocampal axis is needed for effective spatial learning (Loureiro et al., 2012). However, most research studies on hippocampus focus on its dorsal pole. Here, it is well documented that the dorsal hippocampus plays an important role in visuospatial learning, navigation, object memory, as well as the updating of spatial information (Strange et al., 2014; Zemla and Basu, 2017). Moreover, it has been reported that the dorsal hippocampus is more engaged during decision-making and complex cognition within a spatial behavioral task than the ventral hippocampus, although both structures are engaged and active (Schmidt et al., 2013). This would suggest that some degree of functional specialization occurs along the dorsoventral hippocampus with regard to spatial information processing. We observed a dorsoventral gradient of nuclear Homer1a expression triggered by spatial learning, in line with the observations of others using a different IEG (zif268) and different spatial learning conditions (Schmill et al., 2023).

The precision of the dorsal hippocampus in processing and encoding temporal, contextual, predictive, and dimensionality-related aspects of space has been demonstrated by a multitude of studies (see Kinsky et al., 2018; Chockanathan and Padmanabhan, 2021; Centofante et al., 2023; Gonzalez et al., 2025). However, in addition to temporal and spatial information, the dorsal hippocampus also processes non-spatial aspects of experience (Eichenbaum, 2016; Sanders et al., 2019) a process that comprises the fundament of episodic learning and for which the dorsal hippocampus possesses all required attributes (Takahashi, 2018; Sugar and Moser, 2019).

The intermediate hippocampus has been ascribed roles in rapid experience encoding and experience-dependent behavioral flexibility (Bast, 2007; Bast et al., 2009; Jarrard et al., 2012), fulfilling functions that are distinct from dorsal or ventral hippocampus (Whitley et al., 2024). The intermediate hippocampus has also been proposed to combine visuospatial information encoding with sensitivity to emotional context (Hwang et al., 2024; Troha et al., 2025). Inactivation of the intermediate hippocampus does not affect memory (at least in the forms of inhibitory avoidance, spatial alternation, or temporal order memory) that is dependent on dorsal and/ or ventral hippocampal information processing, but prevents rapid place learning and mnemonic control of future behavior, suggesting that the intermediate hippocampus may fulfill a unique role in the processing and encoding of changing contingencies within associative learning (Whitley et al., 2024). In the present study, we only detected information encoding in the dCA1 as a result of novel *microscale* spatial content learning. This finding aligns with reports that that dorsal and intermediate CA1 differ in spatial firing characteristics, but respond similarly to different types of novelty (spatial, social/odor; Lee et al., 2025), but also suggests that this functional alignment may be constrained to dorsal dCA1, that in turn is putatively involved in the integration of both spatial and non-spatial information.

Microscale spatial content learning requires catecholaminergic input to the hippocampus (Lemon and Manahan-Vaughan, 2006, 2012; Kemp and Manahan-Vaughan, 2008a; Hagena and Manahan-Vaughan, 2025). Interestingly, the intermediate CA1 exhibits higher expression of noradrenergic α_2_-receptors, compared to the dorsal and ventral hippocampus (Lothmann et al., 2021). Activation of these receptors mediates anxiolysis (Harvey et al., 2021), suggesting that the intermediate hippocampus might act as an interface that counterbalances negative experience-encoding by the ventral hippocampus (Bannerman et al., 2004; Beyeler et al., 2016). Additionally, it also encodes motivational significance of place and reward location (Jin and Lee, 2021; Jarzebowski et al., 2022). In the present study, animals were well-habituated to the experimenter and to the test chamber. We cannot rule out that, if the experimental conditions were more emotive or stressful, a different engagement of the iDG would have occurred. For example, novel learning about macroscale or microscale spatial content features promotes the expression of short-term synaptic plasticity in the intermediate DG (Kenney and Manahan-Vaughan, 2013), whereas in the dorsal hippocampus *only* macroscale spatial content learning promotes synaptic plasticity, in DG that in this case lasts for over 24 h in rats (Kemp and Manahan-Vaughan, 2008b). This suggests that both the thresholds for, and specificity of, spatial content learning in the dorsal and intermediate hippocampus may be distinct.

Historically, the ventral hippocampus was viewed as a structure that was functionally distinct from the dorsal pole (Moser and Moser, 1998), serving to process and integrate emotional experience, stress and affect into associative experiences (Fanselow and Dong, 2010; Maggio and Segal, 2012). However, evidence has been provided that the ventral hippocampus possesses place cells (Jung et al., 1994). These exhibit a lower spatial selectivity (Jung et al., 1994; Keinath et al., 2014) and larger place fields (Kjelstrup et al., 2008) than detected in the dorsal hippocampus. Studies in human subjects have led to the proposal that the ventral hippocampus (referred to as anterior hippocampus in primates/humans) supports the generation of ‘gist-like’ or global memory representations, whereas the dorsal (posterior) hippocampus is responsible for the building of detail-rich representations (Poppenk et al., 2013). However, large place fields may confer the ventral hippocampus with the ability to link experiences that are both temporally and spatially distant from one another (Buzsáki, 2010; George et al., 2023), a process that is not only needed for effective cognitive generalization (Zeithamova et al., 2012), but also for the linking of remote memories to recent experience (Eichenbaum, 1999).

The ventral hippocampus might thus, play an important role in spatial memory processed by hippocampal-cortical circuits by supporting the representation of temporally linked contextual information. Clues to this role are provided by its unique anatomical connectivity with (sub) cortical structures. The ventral two-thirds of the hippocampus possesses reciprocal connections with the amygdala, nucleus accumbens and hypothalamus, and may function as an interface between subcortical and associative cortical areas (Pikkarainen et al., 1999; Chiba, 2000; Pitkänen et al., 2000; Petrovich et al., 2001; Felix-Ortiz and Tye, 2014; Ritger et al., 2024). This points toward a role for the ventral hippocampus in the integration of emotion and affect into associative experience (Bannerman et al., 2004). Differentiated outputs of the dorsoventral hippocampus support this possibility: for example, whereas neurons of the dorsal CA subfields connect *indirectly* to both dorsal and ventral regions of the prefrontal cortex (PFC; Messanvi et al., 2023), neurons in ventral CA1 and ventral subiculum project *directly* to the ventral PFC (vPFC, Swanson, 1981; Jay and Witter, 1991; Cenquizca and Swanson, 2007; Messanvi et al., 2023). The vPFC has been ascribed multifaceted roles in decision-making, social cognition, emotional regulation and self-related information processing (D’Argembeau, 2013; Hiser and Koenigs, 2018). Strikingly, it is activated both by affective outcomes, and the stimuli that predict those outcomes (Eisenberger et al., 2003). The ventral hippocampus has been reported to integrate broader environmental features and support experience generalization (Keinath et al., 2014; McKenzie et al., 2016). The strong monosynaptic connections from the ventral hippocampus to the vPFC may thus, provide spatiotemporal contextual information that could enable the activation of context-appropriate memory retrieval rules, and related predictions within prefrontal circuits (McKenzie et al., 2016). Another interesting feature of the ventral hippocampus is its distinct topographical projection to the MEC (Ohara et al., 2023): the dorsal CA1/dorsal subiculum sends projections uniquely to layer 5b of dorsal MEC, which in turns innervates CA1-projecting layer 3, whereas the ventral CA1/ dorsal subiculum projects to layer 5b of *both* dorsal and ventral MEC, as well as to layer 5a of ventral MEC, which in turn project to telencephalic structures (Ohara et al., 2023). The distinct afferent and reciprocal connectivity of the hippocampus with the above-mentioned (sub)cortical structures may also explain why the ventral hippocampus engages in visuospatial information encoding, if motivation (reward), goals, or task complexity are a component of the experience (Ruediger et al., 2012; Schumacher et al., 2016; Contreras et al., 2018; Thonnard et al., 2021; Décarie-Spain et al., 2022). In line with this, it has been reported that the ventral hippocampus is required for predictive learning and decision-making about the spatial location of aversive experience (Ito and Lee, 2016; Malagon-Vina et al., 2023).

The fact that neurons in ventral CA subfields were activated only by novel macroscale spatial content learning might be explained by the nature of the landmark objects used in the experiment, given that they are directly visible and larger than objects used in the microscale experiment. Thus, they may have resulted in greater physical contact during exploration and thus activated ventral hippocampal-cortical/subcortical circuits for behavior control, i.e., motivation, goal-oriented exploration and behavioral flexibility for switching task in spatial learning (Brady, 2009; Riaz et al., 2017; Torres-Berrío et al., 2019; Hasantash et al., 2025). Inclusion of physical features to an environment increase place field activity (Burke et al., 2011) and activate hippocampal vector cells (Deshmukh and Knierim, 2013). Behavioral studies also showed that both dorsal and ventral hippocampus are involved in adjusting navigation strategy in obstacle-rich environment (Contreras et al., 2018). This kind of processing requires the support of the prefrontal cortex for path planning and the nucleus accumbens for behavior control (Poucet et al., 2004; Biane et al., 2025). Bearing in mind the strong monosynaptic projections of the ventral hippocampus to the PFC and nucleus accumbens (Phillips et al., 2019; Zhou et al., 2020; Cernotova et al., 2021), we propose that the proximal CA3 of ventral HPC supports the encoding of item identity (“what” information), whereas the ventral CA subfields encode both “what” and “where” information, albeit in a different manner compared to dorsal CA. This interpretation correspond with findings by others that neurons in the ventral CA subfields encode spatial context by integrating both allocentric and egocentric representations, thereby integrating motivation and behavior flexibility into spatial representations (Gofman et al., 2019; LaChance et al., 2019; Cernotova et al., 2021; LaChance and Taube, 2022).

### 4.3 Multisensory considerations

This study, and the discussion of the findings of others detailed above, focused on the involvement of specific hippocampal regions in visuospatial content encoding. This kind of information processing has been reported to facilitate the expression of hippocampal synaptic plasticity (Hagena and Manahan-Vaughan, 2024) as well as engage or modify place cell activity (Nagelhus et al., 2023). Far fewer studies have addressed the extent to which the hippocampus uses spatial information from other sensory modalities to generate representations and memory of spatial experience. With regard to the dorsal hippocampus, it has been shown that it can use largely monosensory olfactospatial and audiospatial information to generate stable place fields (Save et al., 2000; Zhang and Manahan-Vaughan, 2015; Aronov et al., 2017; Fischler-Ruiz et al., 2021; Dolón Vera et al., 2024; Simoes De Souza et al., 2024; Robson et al., 2025) and can also use (somatosensory) path integration to navigate space in darkness (McNaughton et al., 1996; Zhang et al., 2014). However, if given a choice, rodents prefer to rely on distal visuospatial cues to generate place representations (Save et al., 2000; Draht et al., 2017). Synaptic plasticity in the dorsal hippocampus that is facilitated by spatial learning can be enabled by novel visuospatial (Kemp and Manahan-Vaughan, 2008b), audiospatial (Dietz and Manahan-Vaughan, 2017) or olfactospatial information (André and Manahan-Vaughan, 2013), but here too, it has been shown that a multisensory environment is the best substrate for spatial learning (Hagena and Manahan-Vaughan, 2024). This suggests that for the dorsal hippocampus, it is the reliability of the sensory statistics and not the modality *per se* that determines the extent to which monosensory or multisensory in formation is used as a substrate for spatial learning.

Little is known about how the intermediate and ventral hippocampus utilize non-visual information to map or record spatial experience. However, synaptic plasticity in the intermediate dentate gyrus is facilitated by exposure to novel visuospatial information (Kenney and Manahan-Vaughan, 2013) and context-dependent olfactory learning requires engagement of the ventral hippocampus (Levinson et al., 2020). Moreover, exposure to conspecifics triggers string neuronal responses in ventral hippocampus (Rao et al., 2019). These findings may reflect another form of functional specialization that is distinct from the dorsal hippocampus. For example, the ventral, but not dorsal, hippocampus supports working memory of odor experience (Kesner et al., 2011) and engages in olfactory pattern separation (Weeden et al., 2014). In line with this, it is activated by olfactory, but not auditory cues associated with a previous fear conditioning experience, despite subsequent benign habituation to the environment in which the fear conditioning occurred (Sepahvand et al., 2024).

### 4.4 Caveats and limitations

Nuclear expression of IEGs is time-stamped, meaning that each IEG has a specific experience-dependent peak nuclear expression that is unique for a given IEG (Hoang and Manahan-Vaughan, 2025). The IEG then migrates rapidly to the cytoplasm, where it can also be detected (Guzowski et al., 1999; Marrone et al., 2011). Different basal expression levels of IEGs have been reported across different brain structures using the FISH approach implemented here (Bepari et al., 2012). For example, basal Arc expression is notoriously low (Guzowski et al., 1999; Chawla et al., 2005) and basal cFos expression is relatively higher (Hoang and Manahan-Vaughan, 2025). Moreover, basal levels of Homer1a in the CA hippocampus tend to be higher than Arc (Hoang and Manahan-Vaughan, 2025). Thus, it could be the case that the activation threshold for expression of a specific IEG differs depending on the IEG studied. Another important consideration is that the outcome of basal expression of IEG also depends on the specificities of the behavioral experiment. For example, Homer1a expression in the DG, triggered by LM exposure, was higher in the present study compared to a previous report (Hoang et al., 2018), although both studies reported significant effects compared to controls. This might be explained by the fact that in the previous study the test arena had a floor area of 80 cm^2^, whereas in the current study it was 40 cm^2^. Thus, the objects were easier to find in the current study and may have thus appeared more salient. Other factors that can potently affect both basal and experience-dependent IEG expression comprise the degree of habituation of the animals and their affective state during the experiment. Strong cytoplasmic signals arising from stress, or strong arousal immediately prior to the experiment, will render nuclear signal detection more difficult. We took especial care to ensure that our animals were well-habituated both to the test environment and the experimenter, as can be seen by the low cytoplasmic signals in our samples, thereby minimizing these confounds.

In our study, significant elevations of nuclear Homer1a expression were only detected in the dorsal iDG as a result of LM exposure. However, elevations of zif268 using FISH have been reported in dorsal and ventral sDG following spatial learning (Marrone et al., 2011; Satvat et al., 2011). This difference may relate to the abovementioned activation thresholds for a specific IEG. Differences in the experimental design may also underlie the differences in activation responses detected: In the zif268 study, animals engaged in cumulative spatial learning in a plus maze. In another study, where animals were exposed to two geometrically distinct, albeit featureless environments, FISH detected significant increases in nuclear Arc expression in the dorsal sDG, but not iDG (Chawla et al., 2005). Thus, these studies placed different emphasis on ‘what’ and ‘where’ elements of the spatial environment.

In the study by Chawla and colleagues, rats were 9 months old. In the study by Marrone and colleagues, rats were 9–27 months old. Thus, a key determining factor may also be age: the rats used in our study were 7–9 weeks old, and neuromodulation that boosts information encoding in the DG declines rapidly with age (Twarkowski and Manahan-Vaughan, 2016). Furthermore, hippocampal encoding of spatial experience becomes more general and context-invariant with increasing age (Barnes et al., 1997; Crown et al., 2022). The aging hippocampus is also poorer at integrating landmark information into spatial representations (Barnes et al., 1997).

Another possibility is that the iDG is specialized toward detecting pattern completion in terms of spatial content, whereas sDG responds to more generalized spatial change, as occurs when the environment itself changes in dimension, relationship to the distal environment, or overall physical and ambient characteristics of the environment, as was tested in studies on aged rats (Chawla et al., 2005; Marrone et al., 2011; Satvat et al., 2011). Finally, evidence in favor of the validity of our iDG findings comes on the one hand, from our own observation that the proximal CA3 was activated by the same macroscale spatial exploration, and on the other hand by a study that reported that neuronal activation of iDG and pCA3 occurred in the circumstance of pattern separation (Schmill et al., 2023), in alignment with the functional partnership of the DG and pCA3 structures proposed by others (GoodSmith et al., 2017; Lee and Jung, 2017; Senzai and Buzsáki, 2017).

Previous studies have reported left–right brain asymmetries in rodent hippocampal transcriptomic profiles, synaptic properties, and spatial information processing, particularly within the CA1-CA3 circuitry (Shinohara et al., 2008; Shinohara, 2009; Jordan, 2020; Cholvin and Bartos, 2022; Kubota et al., 2025). Nonetheless, the left and right hemispheres share the same molecular architecture and protein-to-protein networks (Kubota et al., 2025) and immediate early genes form consistent (experience-dependent) central hubs across both hemispheres (Kubota et al., 2025). The brain collection strategy used in the present study was chosen to facilitate accurate anatomical localization and dissection of dorsal versus intermediate/ventral hippocampal subregions especially along the distal-proximal axis. Importantly, the same hemisphere sampling procedure was applied consistently across all experimental groups. Furthermore, our primary comparisons were made *between* experimental groups rather than across hemispheres, the main conclusions of the study are unlikely to be driven solely by hemispheric differences. Nonetheless we cannot exclude that left–right brain asymmetries may have affected our results’ outcome.

## 5 Conclusion

Our current study reports a functional segregation with regard to the novel encoding of spatial content across both the proximodistal and dorsoventral axes of the hippocampus. The dorsal hippocampus exhibits the strongest relative response to both forms of spatial content learning: dCA1 and pCA3 encode microscale aspect of spatial learning, whereas dorsal pCA3 and infrapyramidal DG neurons encode information about novel macroscale experience. Neurons in intermediate CA1 were only recruited by novel exposure to microscale spatial information. By contrast, ventral CA1 and pCA3 encode macroscale spatial experience.

In summary, our data, interpreted in light of the findings of others, indicate that the dorsoventral hippocampus works as a unit to generate temporally, spatially and contextually distinct associative memory representations. This occurs by means of rapid detail-rich information encoding (dorsal), flexible response outcomes (intermediate), as well as the linking of remote experience during cumulative emotionally nuanced learning (ventral). The risk of overlapping, or similar, experiences becoming blurred into a common memory is circumvented by a unique form of information processing in the dorsal iDG, that may correspond to the proposed role of the DG in pattern separation (Kesner, 2013; GoodSmith et al., 2017; Lee and Jung, 2017).

These findings suggest that, on the one hand, the entirety of the dorsoventral axis engages in spatial information processing, and on the other hand, that a functional differentiation, with regard to the processing of spatial information, occurs along the dorsoventral axis that is likely to support the optimization of cognitive representations by the hippocampus.

## Data Availability

The raw data supporting the conclusions of this article will be made available by the authors, upon reasonable request.

## Footnotes

1
https://fiji.sc/

2
https://qupath.github.io/

3
https://github.com/stardist/stardist
