# Supplementary material for: Discrete and differentiated encoding of distinct components of spatial experience occurs within the proximodistal subfields of the dorsoventral axis of the hippocampus
Source: Front Behav Neurosci. 2026 Aug 7;20:1895371. doi: 10.3389/fnbeh.2026.1895371 (PMC13493596; doi:10.3389/fnbeh.2026.1895371)
Supplement: Supplementary file 1 [file Table_1.DOCX]

Supplementary Table 1 **Summary of statistical results (post-hoc test) evaluating changes in nuclear Homer1a expression in hippocampal neurons among novel control, microscale (HBO) and macroscale (LM) spatial learning animals**

|  | Control vs HBO | Control vs LM | HBO vs LM |
| --- | --- | --- | --- |
| dorsal_dCA1 | **0.0304** | 0.2612 | 0.2348 |
| intermediate_dCA1 | **0.0199** | 0.9784 | **0.0164** |
| ventral_dCA1 | 0.6027 | **0.0117** | **0.0028** |
| ventral_pCA1 | 0.5541 | **0.046** | **0.0106** |
| dorsal_pCA3 | **0.004** | **<0.0001** | 0.1984 |
| ventral_pCA3 | 0.9025 | **0.0207** | **0.028** |
| dorsal_iDG | 0.6644 | **<0.0001** | **<0.0001** |

The table reports the outcome of post hoc statistical outcomes, obtained using Fisher’s LSD test after two-way ANOVA comparing nuclear Homer1a expression among three groups (control, HBO, LM). Only hippocampal subregions that exhibited a significant behavior task related Homer1a expression effect relative to the control condition are shown. Statistically significant differences (p < 0.05) are highlighted in bold.

Abbreviations: HBO: holeboard, containing objects; LM: landmarks; dCA1: distal CA1; pCA1: proximal CA1; pCA3: proximal CA3; iDG: infrapyramidal blade of dentate gyrus.
